# Management of Foreign Bodies in the Ear, Nose and Throat in Pediatric Patients: Real-Life Experience in a Large Tertiary Hospital

**DOI:** 10.7759/cureus.30739

**Published:** 2022-10-26

**Authors:** Antonella Loperfido, Fulvio Mammarella, Cristina Giorgione, Alessandra Celebrini, Gilberto Acquaviva, Gianluca Bellocchi

**Affiliations:** 1 Otolaryngology Unit, San Camillo Forlanini Hospital, Rome, ITA

**Keywords:** paediatric otolaryngology, children and adolescents, foreign bodies airway, foreign bodies in ear or nose, medical education and training

## Abstract

Background: Foreign body (FB) injuries occur frequently in children. The aim of this paper is to provide an update on the experience of the Department of Otolaryngology, San Camillo Forlanini Hospital in Rome concerning the management of FB injuries in children.

Methodology: This study was carried out by collecting data from the medical reports of our Pediatric Emergency Room stored between 2007 and 2021. Inclusion criteria were diagnosis of FB in pediatric patients based on the ENT evaluation. Pediatric patients included children and preteens ranging from six months to 15 years.

Results: Between 2007 and 2021, 1,623 cases of FBs in young patients (840 males, 783 females, mean age: 5.5 years) were observed at the Pediatric Emergency Room and treated by the ENT Department. The ear was the most frequently involved site (700 patients), followed by the nose (517 cases), pharynx (319 cases), mouth (76 patients) and airways (11 cases). The most common management strategy was FBs’ removal in the emergency room and home discharge (1,409 patients), 99 cases required outpatient discharge, 64 patients moved away from the Emergency Care refusing treatment, 35 patients were hospitalized, 10 patients refused hospitalization, five were transferred to the pediatric hospital and one died in the emergency room.

Conclusions: A quick diagnosis of FB followed by an effective removal is crucial to avoid injuries and complications. Surveillance registries have a key role in the prevention and management of FB injuries. Moreover, it is necessary to train medical and nursing staff of emergency, pediatric and otolaryngologist departments to best recognize and manage FB injuries.

## Introduction

Foreign body (FB) injuries frequently occur in children, particularly in those between 0 and 3 years of age [1]. Commonly, infants involved are less than three years old because, in this age group, children typically use their mouths to explore objects. Moreover, they lack the cognitive ability to distinguish between an edible object from an inedible one. In addition, babies have difficulties forming a food bolus due to their teeth status, especially the lack of molars, and their swallowing coordination is still poor. Finally, some authors report that another cause of FB injuries can be represented by the tendency to be distracted and to perform more activities together, for example, eating and playing at the same time [2]. In addition, FB injuries often have a non-specific clinical presentation which can lead to delayed recognition and possible complications depending mainly on the type of FB and its anatomical location [3]. In fact, ingestion and aspiration of FBs lead to significant morbidity and mortality due to choking, being the fourth cause of accidental death in children under three years of age and the third in those younger than one year [4].

Italy is a member state of the Susy Safe project (Surveillance System on Foreign Body Injuries in Children), an international surveillance registry for injuries due to organic and inorganic FB inhalation, aspiration, ingestion or insertion in children aged 0-14 to create guidelines for FB injury prevention. Specifically, it represents a surveillance registry for injuries gathering data from all European Union (EU) countries and beyond, in order to provide a risk analysis profile for each of the products causing the injury and an evaluation of how socioeconomic disparities among EU citizens may affect the likelihood of being injured by FB ingestion [5]. This surveillance system represents a systematic data collection for FB injuries in children. It allows to identify the FBs most frequently involved. Specifically, among organic FB, the most dangerous reported in the literature are nuts and seeds, while the non-food objects with the highest risk of injury are those with round shapes, such as balls, marbles and coins [6]. The main aims of the project include providing clinicians with reliable data, fostering dedicated educational projects about safe behavior and active parental guard and involving Consumer Associations and/or National Market Surveillance Authorities in data collection and proper education of consumers [7]. Clinicians are focusing more and more on FB injuries because they are regarded as a public health problem, both for the socioeconomic impact and for the costs to the affected children and their family's quality of life [8]. The aim of this paper is to provide an update on the clinical experience of the Department of Otolaryngology, San Camillo-Forlanini Hospital in Rome concerning the management of FB injuries in children [9].

## Materials and methods

This retrospective cross-sectional study was carried out by collecting data from the medical reports of the pediatric emergency room stored using the computerized system GIPSE (GIPSE® digital records, Rome, Italy) (emergency computerized management system in use at the EDs of the entire Lazio Region) between 2007 and 2021 at the San Camillo-Forlanini Hospital in Rome. Inclusion criteria were as follows: diagnosis of FB in pediatric patients based on the ENT physical examination with the possible addition of fiberoptic laryngoscopy or otomicroscopy, according to the anatomical district involved. Pediatric patients included children and preteens ranging from six months to 15 years.

From each record, we collected information about the patient’s age and sex and the location of FBs. Moreover, we reported the management of each patient by distinguishing the patients who had the FB removed and were discharged at home, those discharged with an outpatient appointment, those who were not compliant and spontaneously moved away from the emergency care and those who were hospitalized, the cases that refused the hospitalization, the patients transferred to the pediatric hospital and those died at the emergency room. Ethical approval was waived because we followed the principles of good clinical practice; furthermore, we accessed the anonymized data stored within the institutional software, therefore fully respecting the General Data Protection Regulation (GDPR). Data from the collected cases were collated and processed using the Data Analysis ToolPak loaded in Excel (Microsoft®, Redmond, Washington, United States) to calculate descriptive statistics.

## Results

**Figure 1:**
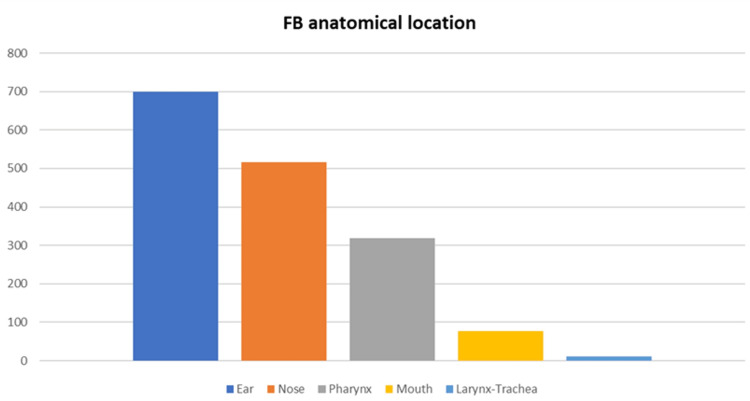
FBs' anatomical location FB: foreign body.

Between January 2007 and December 2021, a total of 1,623 cases of FBs in young patients were observed at the Pediatric Emergency Room of San Camillo-Forlanini Hospital in Rome and treated by the ENT Department. The cohort was constituted of 840 males (51.7%) and 783 females (48.3%) with a mean age of 5.5 years (ranging from six months to 15 years). Regarding the anatomical distribution, the ear was involved in 700 patients (43.1%), the nose in 517 cases (31.8%), the pharynx in 319 cases (19.6%) and 76 patients came to our attention with an FB in the mouth (4.7%) and 11 patients (0.8%) presented the FB in the airways (larynx and trachea). Figure 1 shows the graph of the FBs' anatomical distribution.

**Figure 2:**
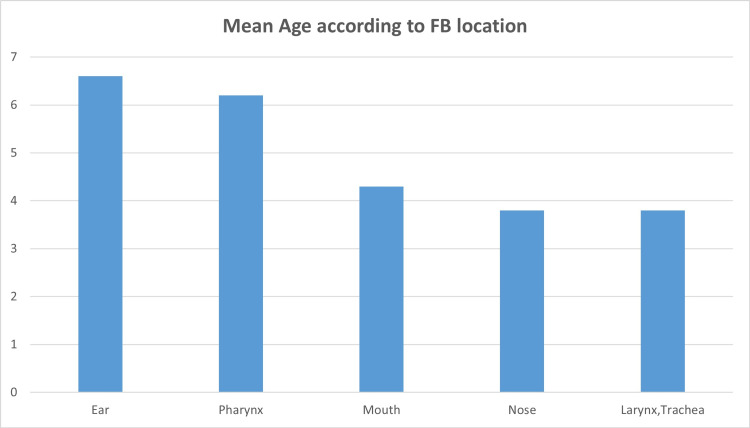
Patients’ mean age according to FB location FB: foreign body.

The mean age of the 700 patients with FB in the ear was 6.6 years (from one year to 14.8 years); the mean age of the 517 patients with FB in their nose was 3.8 years (from six months to 14.9 years); concerning the FBs in the pharynx, the mean age of patients was 6.2 years (from seven months to 15 years); regarding FBs in the mouth, the mean age was 4.3 years (from six months to 14 years) and finally the mean age for FBs in the airways was 3.8 years (from seven months to 14 years). Figure 2 shows patients’ mean age according to FB location.

The most common management strategy was FBs’ removal in the emergency room and home discharge (1,409 patients, 86.8% of all cases), 99 cases required outpatient discharge (6.1%), 64 patients (3.9%) moved away from the emergency care refusing the treatment, 35 patients were hospitalized (2.1%), 10 patients refused the hospitalization (0.6%), five were transferred to the pediatric hospital (0.4%) and one patient died in the emergency room (0.1%). Concerning the inpatients, their mean age was 4.1 years and the most frequently involved district was the nose. Table 1 describes the management outcome for each patient.

**Table 1:** Management outcome *The patient spontaneously moves away from the emergency care.

Location	Number of Patients	Home Discharge	Outpatient Discharge	Non-Compliant Patient*	Hospitalization	Hospitalization Refused	Transfer to the Pediatric Hospital	Death in the Emergency Room
Ear	700 (43.1%)	589	79	17	14	1	0	0
Nose	517 (31.8%)	451	12	32	15	6	1	0
Pharynx	319 (19.6%)	292	7	10	4	2	4	0
Mouth	76 (4.7%)	69	1	5	0	1	0	0
Larynx-trachea	11 (0.8%)	8	0	0	2	0	0	1
Total	1,623	1,409 (86.8%)	99 (6.1%)	64 (3.9%)	35 (2.1%)	10 (0.6%)	5 (0.4%)	1 (0.1%)

## Discussion

FB lesions may occur more frequently in early childhood, usually in the first three years of life, with reported differences between countries: these include one year of age in Puerto Rico, under three years in Argentina and Scandinavia, one to four years of age in Brazil and up to 14 years in Korea [10-14]. The wide range could be explained by taking into account several factors such as the different anatomical districts considered, the level of assistance center (emergency room, pediatric hospital, first- or second-level hospital), the type of medical specialization (pediatric, ENT or pneumological journals) and the lack of a homogeneous data recording system as most publications are from a single monocentric study. Our case series examines the period 2007-2021 for a total of 1,623 patients with an average age of 5.5 years. There was no gender prevalence, thus confirming the available results from previous reports [15].

FB injuries may involve several anatomical sites such as the eyes and the aerodigestive tract. Less frequent FB lesions involve the genitals and rectum [16]. Concerning the head and neck area, FB injuries can affect the ears, nose, mouth, pharynx, larynx, respiratory and digestive tract. FB injuries involving the airways occur more often in infants younger than four years, while insertion of FBs in the ears or nose is more frequently observed in older children [17]. In our experience, the ear is the most involved district; the current result is quite unusual as the involvement of this site is relatively low in several papers [18,19]. The second most frequent district is the nose, with percentages comparable to most of the literature [20,21]. The oropharynx, the third district, was involved with a higher frequency than that reported in other studies and with a peak in the first oral phase (in children one year of age) [18,19]. It is assumable that the ear was the most involved district because here, unlike the airways, the FB does not evoke protective reflexes such as coughing, vomiting or sneezing.

The management of pediatric FBs requires a quick diagnosis to avoid the development of serious complications, in rare cases fatal ones [22]. Although immediate actions are to be undertaken in the event of a life-threatening FB insertion, there are no guidelines on the timing of removal of non-life-threatening FB. It is widely considered appropriate to act quickly, within 24 hours from the FB insertion [23]. The challenge in FB management is therefore to be able to distinguish patients who require immediate intervention from those it can be deferred. Most of the ear and nose FBs can be successfully removed in the emergency department reserving the use of hospitalization for non-compliant patients and the management of FB in the airways [24]. Our experience is in line with these data as most of the FBs were removed in the emergency room.

Ear FB removal is mainly performed by using direct suction, a right-angle hook, or alligator forceps; hospitalization is rarely necessary [25]. Ear FB removal in the emergency department can also be performed with recently described irrigation techniques. Reported contraindications to irrigation are the presence of tympanic membrane perforation, previous ear surgery, the presence of impacted or hygroscopic FBs (for example, popcorn kernels, dried peas and beans) and the presence of signs of otitis externa, which may be exacerbated by irrigation [26]. Similarly, the management of nasal FB is mainly performed in the emergency room by suction and the use of various medical instruments, such as forceps and micro hooks [27]. In a few cases, the removal of nasal FB is performed by the otolaryngologist in the operating room under anesthesia, typically in those cases where FBs are stuck posteriorly inside the nasal fossa or when children are not compliant [28].

Several papers report that pharyngeal FBs represent a common emergency in children and fish bones are the most frequently reported FBs in this anatomical district. Usually, many oropharyngeal FBs in children are dislodged spontaneously due to the powerful movement of the tongue and the large diameter of this district. When no dislodgement is observed, pharyngeal FBs should be removed to avoid complications. The flexible laryngoscope represents a safe and well-tolerated diagnostic tool, and it is worldwide recommended in the upper airways approach. However, when endoscopy results are negative for laryngopharyngeal FBs and symptoms are persistent, a CT scan is suggested to define the exact location of the FB and its relationship to the vital structures in the neck. When the flexible laryngoscope and CT scan are not available, X-ray should be considered for its usefulness in specifically detecting bone chips and metal FBs [29].

Concerning airways, a rigid pediatric bronchoscopy system with optical telescopes and forceps is desirable for tracheobronchial FB aspiration in children [30]. The choice of the type of bronchoscopic technique for FB removal in the airway depends on the type of the object, the severity of the condition and the caliber of the airway. Typically, the procedure is performed under deep anesthesia with spontaneous ventilation, and after an initial diagnostic flexible bronchoscopy to locate the FB in the tracheobronchial tree, the therapeutic procedure can be performed using different types of flexible or rigid forceps, such as rat tooth, alligator or basket forceps [31]. For each anatomical part involved, it is important to perform the most successful method of FB removal, according to clinicians' experience and available instruments.

Injuries and complications due to the insertion of FB in the nasal fossa are more common than in the ear because the FB might grow asymptomatically for several months or years and affect surrounding tissues. Moreover, the nasal FB is likely to move posteriorly up to the upper airways with consequent severe circumstances [32]. The development of complications is statistically related to the time elapsed between the FB insertion and the diagnosis, the age of the patient and the experience of the operator [15]. In nasal cases, the complications described are infection, epistaxis, necrosis and nasal septum perforation [27]. In otological cases, complications are described in up to a quarter of cases and mainly consist of a bruise, laceration, bleeding, otitis externa and traumatic tympanic membrane perforation [33].

In cases involving the upper respiratory tract, the possibility of FB migration with atypical presentations has been described such as prevertebral abscess, local lacerations, perforations or choking which represents the worst complication resulting in death rates even higher than 50% [34]. In such a case, an immediate airway maneuver must be initiated. The most widely known first aid procedure used to treat upper airway obstruction caused by the FB is the Heimlich maneuver. This represents a life-saving technique aimed at expelling obstructing objects performed by placing the arms around the patient and delivering a sharp inward and upward thrust to the abdomen below the rib cage [35]. This maneuver is used to create pressure within the lungs which in turn helps to expel the lodged FB in the airway into the oral cavity which is further removed by hooking movement of fingers or by any instrument [36]. For an improved effectiveness of the maneuver, the child should maintain a prone position while performing the procedure [37]. This life-saving maneuver should always be attempted in such a situation before choosing surgical techniques like tracheostomy [38]. In our experience, the lethal event consequent to choking occurred only once in a case of a laryngotracheal FB.

## Conclusions

The study results emphasize that a correct and quick diagnosis of FB, followed by an effective removal, is crucial to avoid further injuries and complications. Surveillance registries have a key role in the prevention and management of FB injuries. However, educational projects aimed at increasing knowledge on this topic should be implemented for parents. Another important point that emerges from our paper is the urgent need for the development of a shared treatment protocol to reduce improper treatments. Finally, it is necessary to train medical and nursing staff of the emergency, pediatric and otolaryngologist departments to best recognize and manage FB injuries.

## References

[1] Garg J, De Castro F, Puttasidiah P (2022). Ear, nose, and throat foreign bodies in the paediatric population: did the COVID-19 lockdown change anything?. Cureus.

[2] Passali D, Gregori D, Lorenzoni G, Cocca S, Loglisci M, Passali FM, Bellussi L (2015). Foreign body injuries in children: a review. Acta Otorhinolaryngol Ital.

[3] Shlizerman L, Mazzawi S, Rakover Y, Ashkenazi D (2010). Foreign body aspiration in children: the effects of delayed diagnosis. Am J Otolaryngol.

[4] Rodríguez H, Passali GC, Gregori D (2012). Management of foreign bodies in the airway and oesophagus. Int J Pediatr Otorhinolaryngol.

[5] (2022). The Susy Safe project: Surveillance system on foreign body injuries in children. https://www.susysafe.org/index.php?lang=en.

[6] Lorenzoni G, Umihanić S, Azzolina D, Manza E, Brkić F, Gregori D (2018). A novel approach for comparing patterns of foreign body injuries across countries: a case study comparing European Countries and Bosnia and Herzegovina. Int J Pediatr Otorhinolaryngol.

[7] Rodríguez H, Cuestas G, Botto H, Nieto M, Cocciaglia A, Passali D, Gregori D (2016). Complications in children from foreign bodies in the airway. Acta Otorrinolaringol Esp.

[8] Pecorari G, Tavormina P, Riva G, Landolfo V, Raimondo L, Garzaro M (2014). Ear, nose and throat foreign bodies: the experience of the Pediatric Hospital of Turin. J Paediatr Child Health.

[9] Bellocchi G, Acquaviva G, Giammona Indaco F, Eibenstein A (2020). Foreign bodies in the pediatric age: the experience of an Italian tertiary care hospital. Acta Biomed.

[10] Díaz GA, Valledor L, Seda F (2000). Foreign bodies from the upper-aerodigestive tract of children in Puerto Rico. Bol Asoc Med P R.

[11] Chinski A, Foltran F, Gregori D, Ballali S, Passali D, Bellussi L (2012). Foreign bodies in children: a comparison between Argentina and Europe. Int J Pediatr Otorhinolaryngol.

[12] Sinikumpu JJ, Serlo W (2017). Confirmed and suspected foreign body injuries in children during 2008-2013: a hospital-based single center study in Oulu University Hospital. Scand J Surg.

[13] Figueiredo RR, de Azevedo AA, de Avila Kós AO, Tomita S (2008). Complications of ENT foreign bodies: a retrospective study. Braz J Otorhinolaryngol.

[14] Kim SY, Park B, Kong IG, Choi HG (2016). Analysis of ingested foreign bodies according to age, type and location: a retrospective observational study. Clin Otolaryngol.

[15] Cetinkaya EA, Arslan İB, Cukurova İ (2015). Nasal foreign bodies in children: types, locations, complications and removal. Int J Pediatr Otorhinolaryngol.

[16] He S, Zuo ZL (2018). Different anatomical sites of the foreign body injury with 2999 children during 2012-2016. Chin J Traumatol.

[17] Gregori D (2006). The Susy Safe Project: a web-based registry of foreign bodies injuries in children. Int J Pediatr Otorhinolaryngol.

[18] Sebastian van As AB, Yusof AM, Millar AJ (2012). Food foreign body injuries. Int J Pediatr Otorhinolaryngol.

[19] Slapak I, Passali FM, Gulati A (2012). Non food foreign body injuries. Int J Pediatr Otorhinolaryngol.

[20] Endican S, Garap JP, Dubey SP (2006). Ear, nose and throat foreign bodies in Melanesian children: an analysis of 1037 cases. Int J Pediatr Otorhinolaryngol.

[21] Sarafoleanu C, Ballali S, Gregori D, Bellussi L, Passali D (2012). Retrospective study on Romanian foreign bodies injuries in children. Int J Pediatr Otorhinolaryngol.

[22] Laya BF, Restrepo R, Lee EY (2017). Practical imaging evaluation of foreign bodies in children: an update. Radiol Clin North Am.

[23] Berkowitz RG, Lim WK (2003). Laryngeal foreign bodies in children revisited. Ann Otol Rhinol Laryngol.

[24] Kwon B, Choi Y, Kim SK, Hong SJ, Kim YB, Hong SM (2022). Ear, nose, and throat foreign bodies in children: a retrospective study. Children (Basel).

[25] Lou Z (2021). The outcome and complication of endoscopic removal of pediatric ear foreign body. Int J Pediatr Otorhinolaryngol.

[26] Acquaviva G, Loperfido A, Giorgione C, Bellocchi G (2022). Ear foreign bodies removal in the pediatric emergency department: tricks of the trade. Ital J Emerg Med.

[27] Abou-Elfadl M, Horra A, Abada RL, Mahtar M, Roubal M, Kadiri F (2015). Nasal foreign bodies: results of a study of 260 cases. Eur Ann Otorhinolaryngol Head Neck Dis.

[28] Zavdy O, Viner I, London N, Menzely T, Hod R, Raveh E, Gilony D (2021). Intranasal foreign bodies: a 10-year analysis of a large cohort, in a tertiary medical center. Am J Emerg Med.

[29] Huang Z, Li P, Xie L, Li J, Zhou H, Li Q (2017). Related factors of outcomes of pharyngeal foreign bodies in children. SAGE Open Med.

[30] Black RE, Johnson DG, Matlak ME (1994). Bronchoscopic removal of aspirated foreign bodies in children. J Pediatr Surg.

[31] Rodrigues AJ, Scussiatto EA, Jacomelli M (2012). Bronchoscopic techniques for removal of foreign bodies in children's airways. Pediatr Pulmonol.

[32] Yasny JS (2011). Nasal foreign bodies in children: considerations for the anesthesiologist. Paediatr Anaesth.

[33] Prasad N, Harley E (2020). The aural foreign body space: a review of pediatric ear foreign bodies and a management paradigm. Int J Pediatr Otorhinolaryngol.

[34] Igarashi Y, Norii T, Sung-Ho K (2019). New classifications for life-threatening foreign body airway obstruction. Am J Emerg Med.

[35] Sternbach G, Kiskaddon RT (1985). Henry Heimlich: a life-saving maneuver for food choking. J Emerg Med.

[36] Stoner MJ, Dulaurier M (2013). Pediatric ENT emergencies. Emerg Med Clin North Am.

[37] Ojeda Rodriguez JA, Ladd M, Brandis D (2022). Abdominal thrust maneuver. StatPearls [Internet].

[38] Solanki SL, Bansal S, Khare A, Jain A (2011). Heimlich's maneuver-assisted bronchoscopic removal of airway foreign body. Anesth Essays Res.

